# Positron emission tomography as an adjuvant diagnostic test in the evaluation of checkpoint inhibitor-associated acute interstitial nephritis

**DOI:** 10.1186/s40425-019-0820-9

**Published:** 2019-12-21

**Authors:** David Qualls, Harish Seethapathy, Halla Bates, Shahein Tajmir, Pedram Heidari, Paul Endres, Kerry Reynolds, Donald Lawrence, Meghan Sise

**Affiliations:** 1 Department of Medicine, Massachusetts General Hospital, Boston, MA USA; 2 Division of Nephrology, Department of Medicine, Harvard Medical School, Massachusetts General Hospital, 165 Cambridge Street Suite 302, Boston, MA 02114 USA; 3 Harvard Medical School, Boston, MA USA; 4 Department of Radiology, Massachusetts General Hospital, Boston, MA USA; 5 Division of Oncology, Department of Medicine, Massachusetts General Hospital, Harvard Medical School, Boston, MA USA

## Abstract

**Background:**

Acute interstitial nephritis is an immune-related adverse event that can occur in patients receiving immune checkpoint inhibitor therapy. Differentiating checkpoint inhibitor-associated acute interstitial nephritis from other causes of acute kidney injury in patients with cancer is challenging and can lead to diagnostic delays and/or unwarranted immunosuppression. In this case report, we assess the use of ^18^F-flourodeoxyglucose positron-emission tomography imaging as an alternative diagnostic modality in the evaluation of potential acute interstitial nephritis.

**Case presentation:**

A 55-year-old woman with metastatic vulvar melanoma underwent treatment with two cycles of ipilimumab plus nivolumab, followed by seven cycles of nivolumab combined with radiation therapy. During her treatment, she developed non-oliguric acute kidney injury to a creatinine of 4.5 mg/dL from a baseline of 0.5 mg/dL. A clinical diagnosis of acute interstitial nephritis was made, and steroids were initiated, with rapid improvement of her acute kidney injury. Retrospectively, four positron-emission tomography scans obtained for cancer staging purposes were reviewed. We found a markedly increased ^18^F-flourodeoxyglucose uptake in the renal cortex at the time acute interstitial nephritis was diagnosed compared to baseline. In three cases of acute kidney injury due to alternative causes there was no increase in ^18^F-flourodeoxyglucose uptake from baseline.

**Conclusions:**

To our knowledge, this is the first report describing increased ^18^F-flourodeoxyglucose uptake in the renal cortex in a patient with checkpoint inhibitor-associated acute interstitial nephritis. Our findings suggest that ^18^F-flourodeoxyglucose positron-emission tomography may be a valuable test for diagnosing immune-mediated nephritis, particularly in patients where timely kidney biopsy is not feasible.

## Background

Acute interstitial nephritis (AIN) is increasingly being recognized as an immune-related adverse event (irAE) in patients receiving immune checkpoint inhibitor (ICPI) therapy [1]. A recent meta-analysis of 11 clinical trials demonstrated an overall incidence of kidney irAEs of 2.2%, with incidence rising to 4.9% with combination immunotherapy targeting cytotoxic T lymphocyte antigen-4 (CTLA-4) and programmed cell death protein-1 (PD-1) [1]. While relatively uncommon, AIN is an important consideration when evaluating acute kidney injury (AKI) in patients receiving immunotherapy, as early recognition and treatment with steroids can lead to recovery of kidney function; on the other hand, delays in identification and treatment may lead to permanent damage to the kidneys [1]. However, AKI is common in patients with cancer, with a broad differential diagnosis including sepsis, dehydration, nephrotoxin exposure, and metastatic disease leading to urinary tract obstruction [2]. Diagnosing AIN remains a challenge, as clinical features, laboratory testing, and conventional imaging do not reliably distinguish AIN from other common causes of AKI [3–6]. Biopsy remains the gold standard, but is invasive and carries risks of bleeding, and is often delayed by the use of aspirin and anticoagulants in these patients [7–9]. At the same time, empiric management of AIN with corticosteroids without a definitive diagnosis may lead to inappropriate interruption or discontinuation of cancer immunotherapy, and may compromise the efficacy of cancer treatment in these patients [10].

With the rapidly expanding FDA approval of these agents, establishing reliable noninvasive diagnostic testing strategies for the evaluation of AKI in patients on immunotherapy is of paramount importance. One consideration is the use of ^18^F-flourodeoxyglucose positron emission tomography-computed tomography scan (FDG PET-CT). While most commonly utilized for the staging of malignancies, FDG PET-CT has also been used to identify other inflammatory conditions including large-vessel vasculitis, sarcoidosis, and various infections [11]. A recent case series described PET scans in two cases of AIN, noting elevated ^18^F-flourodeoxyglucose (FDG) uptake in the renal cortex for both patients, providing some precedent that FDG PET-CT may be a useful adjuvant diagnostic test in the evaluation of AIN [12]. Anecdotal evidence supporting these findings in 3 other biopsy-proven AIN cases has been reported [6].

In this case report, we discuss a patient with metastatic vulvar melanoma on immunotherapy who developed ICPI-related AIN. Using serial images, we present the evolution of her AIN as seen through FDG uptake in the renal cortices. In patients for whom there is diagnostic uncertainty and a kidney biopsy is not clinically tenable, FDG PET-CT may represent an additional tool for the evaluation of AIN.

## Case presentation

### Clinical course

**Fig. 1:**
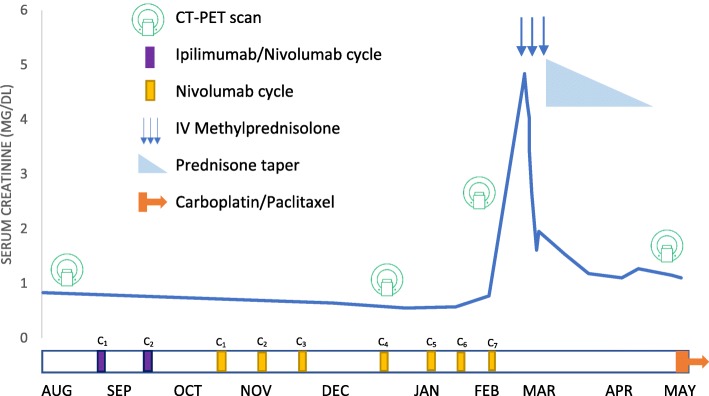
Clinical course of immune checkpoint inhibitor-related acute interstitial nephritis, response to therapy, and timing of PET-CT scans. The patient tolerated 2 cycles of combined ipilimumab and nivolumab followed by 7 cycles of nivolumab monotherapy with stable renal function. After the seventh cycle of nivolumab, an AKI rapidly developed reaching a peak of 4.84 mg/dL. After failing to improve with IV hydration, she was treated with methylprednisolone 500 mg IV daily for 3 days (arrows) followed by a prednisone taper, with rapid improvement in creatinine. Due to progression of disease and AIN, immunotherapy was discontinued indefinitely, and she began therapy with carboplatin and paclitaxel. Notably throughout the course, 4 PET-CT scans were performed, including two prior to the patient’s AKI, one during the AKI, and one following recovery of renal function. Abbreviations: *PET-CT* Positron emission tomography – computed tomography, *AKI* acute kidney injury, *AIN* acute interstitial nephritis

A 56-year-old woman was diagnosed with vulvar melanoma and pulmonary, hepatic and pelvic nodal metastases (Fig. 1). She initially underwent two cycles of combination ipilimumab (anti-CTLA-4) and nivolumab (anti-PD-1) with staging CT scans one month later showing progression of metastatic disease. This prompted a transition to nivolumab monotherapy combined with palliative radiation; ipilimumab was discontinued due to its toxicity with concurrent radiation. She underwent 7 additional cycles of nivolumab, 24 Gy to the vulvar mass and pelvic adenopathy, and 72 Gy total to tibial, T-spine and sacral lesions without apparent complication. Her 8^th^ cycle of nivolumab was delayed for two weeks due to subclinical elevations in liver transaminases. When she re-presented to resume immunotherapy, the patient reported one week of fatigue, nausea and vomiting, along with cough and congestion. Laboratory studies performed at the time were notable for an AKI with a serum creatinine of 4.5 mg/dL, up from a baseline of 0.5 mg/dL. She was admitted to Massachusetts General Hospital for further evaluation and management.

On admission she was given two liters of normal saline for possible dehydration, and despite this, her creatinine rose to 4.8 mg/dL. She was noted to be non-oliguric with a urine output of over 2 liters on the first day of hospitalization. Her other medications included omeprazole 40 mg daily and aspirin 325 mg daily, along with oxycontin and oxycodone for cancer pain. She denied use of any over-the-counter medications, specifically use of any nonsteroidal anti-inflammatory drugs (NSAIDs) or herbal medications.

Initial laboratory testing is shown in Table 1. Urinalysis showed only 1+ blood and 1+ leukocyte esterase, and was otherwise unremarkable; sediment was bland without erythrocytes, nucleated cells, or cellular casts. Urine protein-creatinine ratio was mildly elevated at 331 mg/g and microalbumin/creatinine ratio was also mildly elevated at 72 mg/g. Serologic workup for other etiologies of AKI was negative (Table 1). Ultrasound of her kidneys performed on admission was notable for increased echogenicity of the kidney parenchyma bilaterally, but with no evidence of hydronephrosis or obstruction. Her right kidney measured 11.8 cm and left kidney measured 12 cm. Because she was taking aspirin 325mg daily until the day of admission, kidney biopsy could not be safely performed on presentation.

**Table 1:** Laboratory data obtained during admission for acute kidney injury

Lab	Reference Range	Result
Sodium (mmol/L)	135–145	126
Potassium (mmol/L)	3.4–5.0	3.6
Chloride (mmol/L)	98–108	87
Carbon Dioxide (mmol/L)	23–32	24
BUN (mg/dL)	8–25	43
Creatinine (mg/dL)	0.60–1.50	4.5
Glucose (mg/dL)	70–110	158
Calcium (mg/dL)	8.5–10.5	7
Albumin (g/dL)	3.3–5.0	3.4
Total Protein (g/dL)	6.0–8.3	7.3
Alkaline Phosphatase (U/L)	30–100	124
Bilirubin (Total) (mg/dL)	0.0–1.0	0.2
AST (SGOT) (U/L)	9–32	15
ALT (SGPT) (U/L)	7–33	6
TSH (uIU/mL)	0.40–5.00	4.2
ESR (mm/hr)	0–20	104 mm/hr.
CRP (mg/L)	< 8.0	102.1 mg/L
Total Complement (U/ml)	42–95	71.3
C3 (mg/dL)	81–157	124
C4 (mg/dL)	12–39	36
ANA	Negative	Positive at 1:40, negative at 1:80
dsDNA Antibody	Negative	Negative at 1:10
ANCA	Negative	Negative

Abbreviations:*BUN* Blood urea nitrogen, *AST* Aspartate aminotransferase, *ALT* Alanine aminotransferase, *TSH* Thyroid-stimulating hormone, *ESR* Erythrocyte sedimentation rate, *CRP* C-reactive protein, *ANA* Antinuclear antibody; *dsDNA* Double-stranded DNA, *ANCA* Antineutrophil cytoplasmic antibody.

Given the high probability of ICPI-related AIN and inability to perform kidney biopsy in a timely manner, she was started on pulse dose intravenous methylprednisolone 500 mg daily for 3 days, followed by a prednisone taper starting at 40 mg daily. Her home omeprazole was changed to an H2 blocker, given its known association with AIN [13]. Her creatinine quickly improved from a peak of 4.8 mg/dL on the day steroids were initiated, to 2.7 mg/dL on the fourth day of steroid treatment (Fig. 1). One month after steroid initiation, her creatinine had stabilized at a new baseline of 1.1–1.2 mg/dL. Given the lack of clinical response of her metastatic disease to ICPI-therapy, she was not re-challenged, and was instead treated with paclitaxel and carboplatin.

### Evaluation of FDG PET-CT scan data

**Fig. 2:**
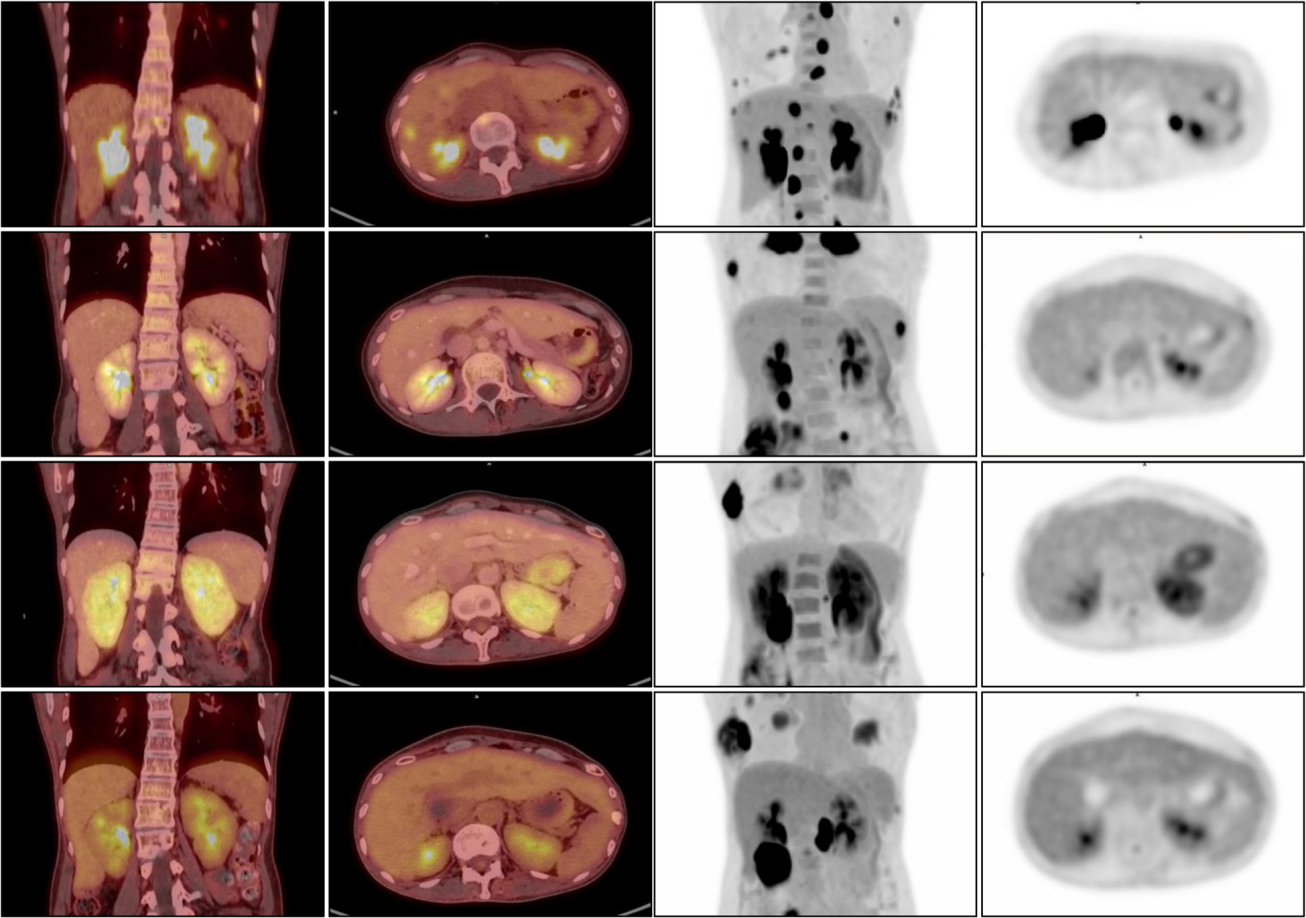
PET-CT scans obtained before and during patient’s treatment course. The initial scan (**a**) was obtained prior to immunotherapy treatment, and the subsequent scan (**b**) was performed after initiation of immunotherapy. Both (**b**) and (**b**) were performed at baseline renal function, and results of the scans show normal renal parenchymal FDG uptake and expected excretion of FDG into the renal pelvises. The third scan (**c**) was obtained during the patient’s AKI, and demonstrates markedly increased FDG uptake within the renal parenchyma compared to (**a**) and (**b**). Following steroid treatment and recovery of renal function, a fourth scan (**d**) was obtained, showing a return to baseline renal parenchymal FDG uptake, consistent with resolution of interstitial inflammation. Abbreviations: *PET-CT* positron emission tomography–computed tomography, *FDG* fluorodeoxyglucose

We noted that 10 days prior to the diagnosis of the patient’s AKI, she had an FDG PET-CT performed for routine cancer staging. At the time of the scan, the contrast-enhanced CT portion of the exam demonstrated multifocal patchy bilateral heterogeneous enhancement in her kidneys, without mass lesions or evidence of obstruction; however, at the time, elevated FDG uptake in the renal cortex was not specifically interpreted. Retrospectively, the FDG PET-CT scans were re-evaluated by a nuclear radiologist (PH) in light of the clinical suspicion for ICPI-AIN. The scans demonstrated increased FDG uptake in the renal cortices bilaterally with a maximum standardized uptake value (SUVmax) of 4 at the time of AKI **(**Fig. 2**)**. These findings were compared to the FDG uptake in PET-CT scans performed on the same patient on three other occasions: prior to initiation of checkpoint inhibitor therapy, after ICPI initiation but before onset of AKI (2 months before AKI), and after recovery from the AKI (3 months after AKI diagnosis). Fig. 1 shows the relationship of FDG PET-CT scans to clinical course of AKI. Compared to baseline and follow-up, the FDG uptake in the renal cortices on the PET-CT scan just before AKI diagnosis had the highest SUVmax **(**Table 2**)**.

**Table 2:** Cortical SUVMax over time in patient with checkpoint inhibitor-related AIN

Time	Clinical Context	SUVMax
0 months	Baseline (prior to ICPI)	2.3
4 months	Post-ICPI initiation, renal function stable	3.4
6 months	During AKI, prior to steroids	4.0
8 months	After steroid treatment and AKI recovery	2.6

Abbreviations: *SUV* Standardized uptake value, *ICPI* Immune checkpoint inhibitor

### Evaluation of FDG PET-CT in non-inflammatory AKI

In order to further evaluate whether enhanced FDG uptake could differentiate AIN from other causes of AKI in patients taking ICPIs, we evaluated 3 patients who had FDG PET-CT performed within 2 weeks of AKI diagnosis and in whom a non-inflammatory etiology of their AKI could clearly be established. Each patient also had a separate PET-CT performed prior to AKI which served as a baseline image. We assessed control and AKI images for SUVMax, with the results demonstrated in Table 3**.** For all 3 patients with non-AIN AKI, including pre-renal azotemia or cardiorenal syndrome, SUVMax at the time of AKI was stable or slightly decreased as compared to baseline FDG PET-CT.

**Table 3:** PET-CT scan findings in patients with hemodynamic (non-inflammatory) AKI

Patient	Etiology of AKI	Baseline SUVMax	SUVMax at time of AKI	Change in SUVMax
1	Cardiorenal AKI from decompensated cirrhosis, atrial mass requiring resection	4	3.8	−0.2
2	Prerenal AKI due to small bowel obstruction	2.8	2.8	0
3	Prerenal AKI due to intraabdominal sepsis	3.1	2.9	−0.2

Comparison of cortical SUVMax before and during AKI for 3 patients undergoing checkpoint inhibitor therapy who had PET performed at the time of AKI. All patients had AKI attributed to clear hemodynamic causes and the AKI resolved without the need for corticosteroids. Patients had PET scans within 0–12 days of AKI

Abbreviations: *AKI* Acute kidney injury, *SUV* Standardized uptake value, *AIN* Acute interstitial nephritis

## Discussion and conclusion

In this case report we describe a patient with a clinical diagnosis of ICPI-AIN, with PET-CT showing increased FDG uptake in the renal cortex at the time of ICPI-AIN. Checkpoint inhibitor-related AIN is characterized by a lymphocyte-predominant infiltrate with varying degrees of plasma cells and eosinophils [1]. Such metabolically active infiltrates readily take up FDG and can be appreciated on FDG PET-CT scan [14]. The described case supports the hypothesis that the inflammatory infiltrate in AIN drives FDG uptake, as our patient with ICPI-AIN had increasing cortical FDG uptake, in contrast to the 3 patients with non-AIN AKI had no change or a decrease in FDG uptake during PET-CT scans obtained at the time of AKI.

Only one other case series by Katagiri et al. has reported on the use of PET-CT scan for evaluating AIN [12]. In this report two patients with drug-induced AIN resulting in oliguric kidney failure were found to have increased kidney parenchymal FDG uptake on PET-CT. A third patient with pauci-immune crescentic glomerulonephritis had PET-CT which showed no parenchymal FDG uptake, suggesting that the enhanced uptake seen in the AIN cases was driven by metabolically active inflammatory cells invading the tubulointerstitial space.

There are key differences between the cases reported by Katagiri et al. and the above report. This is the first reported case of FDG PET-CT detecting changes in checkpoint-associated AIN, as opposed to drug-induced AIN. In the cases reported by Katagiri et al., all patients were oliguric, and scans showed parenchymal FDG uptake without excretion into the renal pelvis. There was concern that in non-oliguric patients, excretion of FDG into the renal pelvis may interfere with the interpretation of renal cortical FDG update. However, our case demonstrates that in a non-oliguric AKI with ongoing active excretion of FDG into the renal pelvis, a pathologically elevated SUVmax within the renal cortex could still be appreciated on PET-CT.

Non-invasive diagnosis of AIN is challenging. The classic clinical criteria, including fevers, arthralgias, and rash, are found in the minority of patients with drug-induced or ICPI-related AIN [1, 3]. Diagnostics including urine chemistries and sediment analysis are unreliable markers for differentiating AIN from other causes of AKI [4–6]. Urine eosinophils are frequently obtained in the evaluation of AIN, but its performance has been unreliable in a number of smaller studies, with the largest study to date finding a sensitivity of 30% and specificity of 68% [4, 6]. A recent study showed that leukocyturia occurs in only half of the patients with checkpoint-related AIN [15]. Moledina and colleagues evaluated if specific T-cell cytokine levels could serve as biomarkers to distinguish AIN from other causes of AKI and found higher levels of urinary tumor necrosis factor-alpha and interleukin-9 in the urine of patients with biopsy proven AIN compared to other kidney diseases [16]. Studies that evaluate these cytokines in patients taking ICPIs are needed. Other imaging modalities, such as gallium scans, have been proposed as an alternative diagnostic strategy in AIN. ^67^Gallium binds to lactoferrin, which is released by infiltrating leukocytes and expressed on the surface of lymphocytes found in the tubular interstitium in AIN, and early studies had suggested that gallium scans were highly sensitive for AIN [17]. However, subsequent studies have found lower sensitivities ranging from 58 to 69% and low specificities of 50–60%, limiting their utility [6].

The only reliable method for diagnosing AIN remains a kidney biopsy, which is often not feasible in patients with advanced malignancies. As in this case, patients are often on aspirin, NSAIDs, or anticoagulation. In such cases, a wash-out period of 7–10 days is recommended to minimize the risk of clinically significant bleeding related to a biopsy [7]. Even in the absence of blood thinners, there is still a ≥ 1% risk of clinically significant bleeding requiring transfusions [8]. Additionally, many patients have absolute or relative contraindications to kidney biopsy, such as having a solitary functioning kidney, morbid obesity, or an inability to hold anti-platelet agents or anticoagulants [9].

Given the risks and potential delays associated with kidney biopsy, clinicians are often left with the dilemma of whether to empirically treat for AIN without a definitive diagnosis. However, a misdiagnosis of AIN and empiric treatment are not without risk, and may compromise treatment of the underlying cancer. While some studies suggest the treatment of irAEs with high-dose steroids do not adversely affect outcomes, their harm has not been definitively ruled out, and one study has shown increased mortality with the use of higher doses of steroids in patients with immune-related hypophysitis [10, 18]. As such, establishing the etiology of AKI in these patients to the greatest extent of certainty possible is vital, and FDG PET-CT may represent an additional tool in determining the etiology.

FDG-PET CT has also been investigated in checkpoint inhibitor-associated gastrointestinal toxicities. Lang et al. prospectively evaluated 100 patients treated with ipilimumab for melanoma, and had PET-CT performed prior to ipilimumab and after 2 and 4 cycles of ipilimumab [19]. They noted a statistically significant correlation between increased FDG uptake throughout the colon and clinical symptoms of colitis; 29 patients developed clinical signs of colitis, and 21 of these patients had increased colonic uptake on PET-CT. The remaining 8 patients with colitis symptoms and negative PET-CT did not have diarrhea at the time PET-CTs were performed. Two separate case reports describe instances of gastroduodenitis and esophagitis/gastritis in patients receiving ICPIs, where FDG PET-CT detected enhanced FDG uptake within the affected organs [20, 21].

As a single case report, significant research is still required to investigate whether FDG PET-CT would be a reliable diagnostic tool for ICPI-AIN. There are other limitations to the utility of FDG PET-CT in general, including its expense and lack of availability at all institutions [22], though PET-CTs are widely available in centers which have an immunotherapy program and patients on ICPIs. Limitations in the resolution of PET-CT may also limit its ability to detect changes in parenchymal FDG uptake; the average renal cortex thickness is approximately 6 mm, and the resolutions of PET scanners can range from < 5 mm for newer models utilizing time-of-flight technology to 10 mm in older models [23]. Variability in PET acquisition protocols, reconstruction algorithms, and the timing and dose of FDG injection may also affect the interpretation of SUVmax, particularly if scans are obtained at separate facilities [24]. We found the comparison with a baseline scan to be extremely helpful, with increasing FDG-avidity from baseline in our patient with checkpoint nephritis, compared to those with other causes of AKI where there was no increase in FDG uptake at the time of AKI.

Other confounders, including kidney neoplasms, metastases, or alternative causes of immune kidney disease, may also interfere with the interpretation of results. As such, FDG PET-CT will need to be used and interpreted in the context of the patient’s renal disease as well as that of the patient’s underlying cancer.

Finally, our case is limited by the fact that the diagnosis of ICPI-AIN was made without kidney biopsy; however, given the clinical features of this case, including enlarged, echogenic kidneys on ultrasound, lack of improvement despite intravenous hydration with normal saline, and the rapid improvement in creatinine after initiating corticosteroids, the treating nephrologist and oncologist were confident in the diagnosis of ICPI-AIN.

Despite these limitations, FDG PET-CT represents a unique opportunity for diagnostic insight in a disease where noninvasive testing is extremely limited. PET-CT has the advantage of being a non-invasive and non-nephrotoxic study if performed without iodinated contrast. In cancer patients specifically, there may be pre-treatment FDG PET-CT scans available for direct comparison, which may assist in diagnostic clarity. As the indications for ICPI therapy expand, the number of cases of AKI with immune-related kidney injury will continue to rise. FDG PET-CT could potentially help differentiate AIN from other causes of AKI, thereby facilitating more accurate and timely diagnosis, and proper treatment. Further evaluation of PET-CT scans performed in patients with AKI on ICPI therapy may yield greater insight into the frequency and pathophysiology of ICPI-associated AIN.

## Data Availability

Not applicable.

## Abbreviations

AIN: Acute interstitial nephritis
AKI: Acute kidney injury
CTLA-4: Cytotoxic T lymphocyte antigen-4
FDG: ^18^F-flourodeoxyglucose
ICPI: Immune checkpoint inhibitor
irAE: Immune-related adverse event
NSAID: Nonsteroidal anti-inflammatory drug
PD-1: Programmed cell death protein-1
PET-CT: Positron emission tomography-computed tomography scan
SUVmax: Maximum standardized uptake value
